# Modified method to improve the design of Petlyuk distillation columns

**DOI:** 10.1186/1752-153X-8-41

**Published:** 2014-06-30

**Authors:** Javier G Zapiain-Salinas, Juan Barajas-Fernández, Raúl González-García

**Affiliations:** 1 Facultad de Ciencias Químicas, Universidad Autónoma de San Luis Potosí, Av. Manuel Nava No.6, Zona Universitaria, San Luis Potosí S.L.P 78210, México; 2 División Académica de Ingeniería y Arquitectura, Universidad Juárez Autónoma de Tabasco, Cunduacán, Tabasco 86690, México

**Keywords:** Distillation, Petlyuk column, Response Surface, Energy integration, Ternary separation

## Abstract

**Background:**

A response surface analysis was performed to study the effect of the composition and feeding thermal conditions of ternary mixtures on the number of theoretical stages and the energy consumption of Petlyuk columns. A modification of the pre-design algorithm was necessary for this purpose.

**Results:**

The modified algorithm provided feasible results in 100% of the studied cases, compared with only 8.89% for the current algorithm. The proposed algorithm allowed us to attain the desired separations, despite the type of mixture and the operating conditions in the feed stream, something that was not possible with the traditional pre-design method. The results showed that the type of mixture had great influence on the number of stages and on energy consumption. A higher number of stages and a lower consumption of energy were attained with mixtures rich in the light component, while higher energy consumption occurred when the mixture was rich in the heavy component.

**Conclusions:**

The proposed strategy expands the search of an optimal design of Petlyuk columns within a feasible region, which allow us to find a feasible design that meets output specifications and low thermal loads.

## Background

Distillation, one of the unit operations employed for separation of vapour-liquid mixtures, is a very energy demanding process. The use of non-conventional distillation columns, such as those suggested by Petlyuk et al. [1], can generate significant savings in energy consumption and thereby significantly reduce the capital costs compared to conventional sequences. The use of thermally coupled columns has decreased the costs of equipment and energy consumption by up to 30% [2-8].

**Figure 1:**
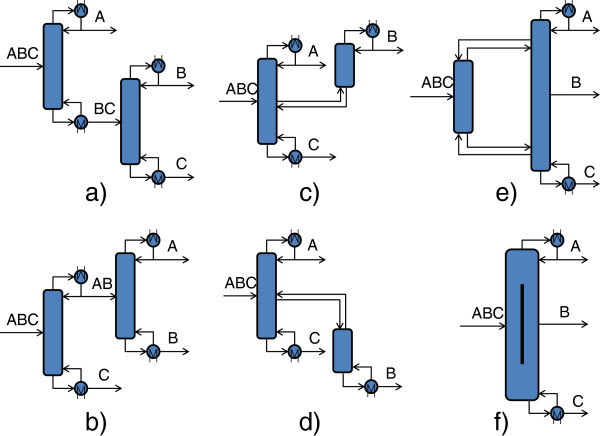
Conventional (a, b) and non-conventional (c, d, e and f) sequences for separating ternary mixtures.

Figure 1 shows examples of separation sequences by distillation, both conventional (a and b) and non-conventional (c, d, e and f), for the separation of ternary mixtures. Configurations c) and d) are employed frequently in the oil industry for the separation of air by cryogenic distillation (for example, a side rectifier is used for the recovery of argon). The e) configuration is a fully thermally coupled column, known as a Petlyuk column [4,5,7-11]. The f) configuration corresponds to the divided wall column [10], also known as Kaibel column; these columns are currently used in the industry [11].

The proposed methods for the design of thermally coupled columns involve a pre-design stage that consists in breaking the system into a set of equivalent columns. The results from the pre-design stage are used to determine the initial design configuration of the Petlyuk column, which must be optimized in order to attain minimum energy consumption.

Other works about the design of Petlyuk-type columns have reported case studies of hydrocarbon mixtures with an equimolar composition [5-8] or with a high concentration of some of the components [12-15]. However, the pre-design method reported fails many times (no convergence to a solution) or yields infeasible solutions (poor quality output streams) when applied to other mixtures or other feed conditions such as non-equimolar mixtures and mixtures in which no single component is more highly represented than others. These facts are demonstrated in this work.

This paper proposes modifying the algorithm used in the pre-design of Petlyuk-type columns for distillation of ternary mixtures so that the pre-design stage is always successful and guarantees feasible results. Separation fractions are established in the equivalent configuration by minimizing the total sum of the theoretical stages and restricting the composition of the output streams to a desired value.

## Methods

The method used for designing Petlyuk-type columns consists of two stages: a) pre-design and b) optimal design.

### Pre-design

**Figure 2:**
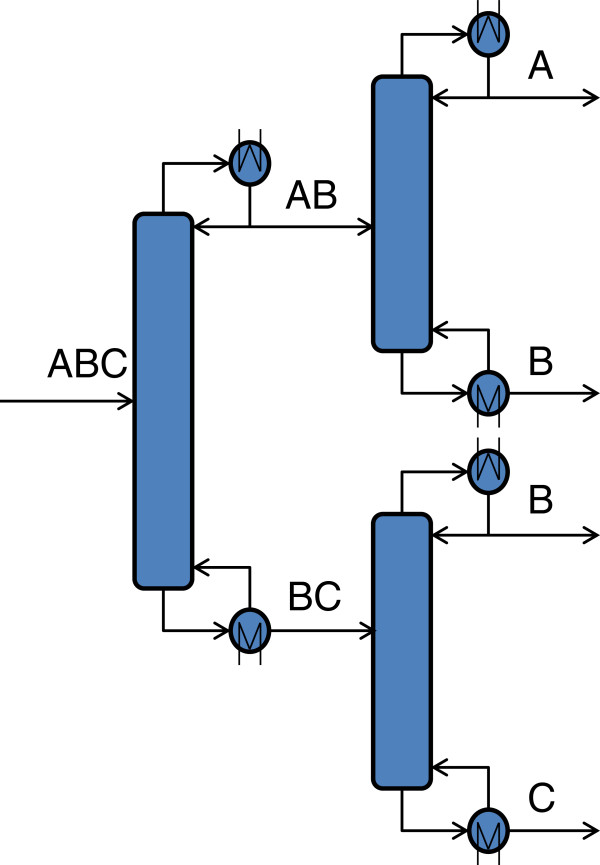
Equivalent configuration of a Petlyuk-type column for separating a ternary mixture.

This stage consists in creating a distillation sequence equivalent to the Petlyuk column. The equivalent configuration for separating three components is shown in Figure 2; it contains a sequence of three columns. Each column of this sequence is designed using the Fenske-Underwood-Gilliland (FUG) procedure for multicomponent distillation with the shortcut method [4,16].

The FUG design method requires knowing the following about each column; a) feeding conditions, (established by the problem to be solved and by the interconnection between columns); b) the operation reflux ratio (fixed to 1.2 times the minimum reflux); c) pressure and type of condenser (fixed according to the FUG method); d) recovery fractions of key components. Conditions (a) to (c) are established in the traditional manner of the FUG method as described by Henley and Seader [16]. However, the manner in which condition (d) is established is where the difference between the traditional pre-design method and the method proposed in this article lies.

**Figure 3:**
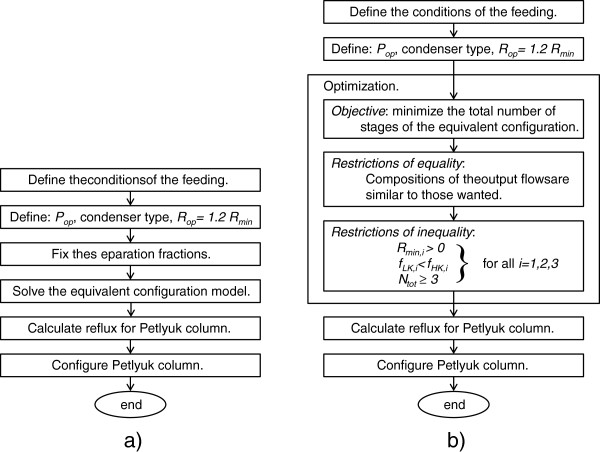
Algorithms of the solution for pre-designing Petlyuk-type columns: a) Traditional method with a fixed fraction; b) method proposed in this work.

In all cases reported in the literature [2,3,13,15,17], the recovery fractions for all columns are set directly, with values ranging from 95% up to 98% in both key components. In the method proposed in this work, the recovery fractions are established by an optimization problem in which the total number of theoretical stages of the equivalent configuration is minimized and the composition of the output streams are restricted in order to reach their desired value. The target function of the optimization problem, which must be minimized, is presented in Equation 1:

(1) $$\begin{array}{l} g_{1}\left(f_{\mathrm{LK},1},f_{\mathrm{HK},1},f_{\mathrm{LK},2},f_{\mathrm{HK},2},f_{\mathrm{LK},3},f_{\mathrm{HK},3}\right)\\ \;=\sum_{i=1}^{3}N_{i}=N \end{array}$$

where *f* is the recovery fraction of key light components (sub-index *LK*), or key heavy ones (*HK*), in the respective column (sub-indexes 1, 2 or 3); *N*_*i*_ is the number of theoretical stages of the column *i* (1, 2 or 3) and *N* is the total number of theoretical stages of the complete system. The problem, set forth with only the objective function, contains 6 degrees of freedom; however, 4 of them are equality restrictions that restrict the composition of the desired product. In this sense, the composition of the light component (component A) in the top product stream of column 2 is established at a desired value of purity; the composition of the intermediate component (component B) in the bottom product stream of column 2, as well as the top product stream of column 3, are established at the same desired value of purity, while the composition of the heavy component (component C) in the bottom product stream of column 3 is established at a desired value of purity. In this work, a purity of 98% was established for each of the products; however, any other value may be applied and the method will still work correctly. This composition allows the streams produced to present a high level of purity. With these equality restrictions, the problem of optimization is left with only 2 degrees of freedom and the feasibility of the solutions is ensured. Inequality restrictions are established in order to ensure that the problem can be solved. In any column, the minimum reflux ratio must be greater than zero, the top recovery fraction of the light key component must be greater than the top recovery fraction of the heavy key component, and must have at least 3 stages. Figure 3 shows the algorithms for the two cases discussed.

It has been found that in the traditional method, in which the separation fractions are specified, the composition of the output streams differs from the desired specifications, mainly when the feed composition is not equimolar. In contrast, the method proposed here ensures that the composition of the output streams is equal to the one specified by the user, even though the composition and thermal conditions of the feed streams vary.

### Optimal design

The information obtained from the equivalent configuration (theoretical stages, molar reflux ratio, interconnection stream flows, flow of each component in each output stream and the position of the feed stage) is used for making the initial estimate in the second design stage of the Petlyuk-type column. At this point, the most valuable information of the proposed pre-design method is the information of the flows of the interconnection streams.

**Figure 4:**
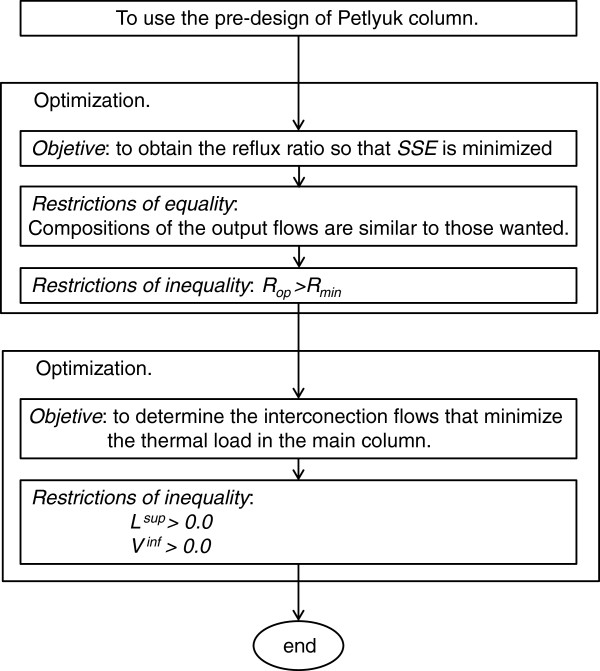
Algorithm for optimal design of Petlyuk-type columns.

Using rigorous methods, it is possible to determine at this stage the operating conditions that are necessary for the Petlyuk-type column to consume a minimum amount of energy. In our work, this stage performed in the same way as reported in the literature [1,7]. The algorithm is represented in Figure 4. The optimal design is developed by performing two optimizations: the reflux ratio of the main column is determined by the first optimization, and the flows of the interconnection streams, which minimize the thermal load of the Petlyuk column are determined by the second one.

The value of the reflux ratio is obtained by minimizing the objective function presented in Equation 2, which represents the sum of the squares of the errors between the calculated and the desired molar flows:

(2) $$\begin{array}{ll} g_{2}\left(\mathrm{ratio}\;\mathrm{of}\;\mathrm{reflux}\right)= & {\left(d_{A}-d_{A}^{*}\right)}^{2}\\ & +{\left(s_{B}-s_{B}^{*}\right)}^{2}+{\left(b_{A}-b_{A}^{*}\right)}^{2} \end{array}$$

where *d*_*A*_ and $d_{A}^{*}$ are the molar flows (calculated and desired) of the A (light) component in the distillation; *s*_*B*_ and $s_{B}^{*}$ are the molar flows (calculated and desired) of the B (intermediate) component in the lateral stream; *b*_*c*_ and $b_{C}^{*}$ are the molar flows (calculated and desired) of the C (heavy) component in the bottom stream. The calculated values refer to those obtained for the Petlyuk-type column and the desired values are those obtained in the pre-design stage.

Once the reflux ratio is obtained, the amount of energy used (*Q*) is minimized using Equation 3, defined as the sum of the absolute values of heat required by the boiler (*Q*_*r*_) and the heat removed in the condenser (*Q*_*c*_); the minimization is achieved by the variation of the interconnection flows (*L*^*sup*^, *V*^*inf*^) of the Petlyuk-type column.

(3) $$g_{3}\left(L^{\text{sup}},V^{\text{inf}}\right)=\left|Q_{r}\right|+\left|Q_{c}\right|=Q$$

The optimal design of a Petlyuk-type distillation column is achieved after carrying out this step.

### Experimental

The cases studied here allow us to show the benefits and problems of both algorithms when using them for pre-designing Petlyuk-type columns, and also for estimating general response surfaces. In all cases, it is taken for granted that the system is working at a constant pressure with a feed flow rate of 45.36 kgmol/h.

In order to show the benefits of the proposed method, only three factors were studied. However, further studies should be done including more factors. The factors studied here are:

a) Mixture type (*M*). Three hydrocarbon ternary mixtures were studied. Two of which have been already mentioned in the literature regarding their separation in Petlyuk-type columns [3,4,17]: n-pentane/n-hexane/n-heptane (*M*_*1*_), n-hexane/n-heptane/n-octane (*M*_*2*_); a third mixture has not been reported yet in the literature: n-hexane/benzene/toluene (*M*_*3*_).

b) Composition of the feed (*Fa*, *Fb* y *Fc*). Where *Fa* is the molar fraction of the most volatile component; *Fb* is the molar fraction of the intermediate volatility component, and *Fc* is the molar fraction of the smallest volatile component. These three quantitative factors represent the composition of a mixture and their sum must be one: *Fa + Fb + Fc = 1*. The composition of each feed was studied within a range of 0.10 to 0.80.

c) The thermal conditions of the feed (*q*). This quantitative factor was studied within a range of 0.00 (saturated vapour) to 1.00 (saturated liquid), that is to say, the mixtures were fed in a liquid–gas state.

In the distillation columns, higher fixed costs are generated by the column size, and higher operating costs are generated by thermal loads. Here we studied the effect caused by the factors mentioned above on the following two response variables:

a) The total number of theoretical stages for the equivalent configuration (*N*); this number is obtained as a result of an optimization carried out in the pre-design stage, Equation 1. The minimum value of this parameter was searched for; the number of theoretical stages accounts for a large percentage of the cost of equipment investment.

b) Thermal load of the Petlyuk column (*Q* in MW), which results from solving the optimization problem proposed in Equation 3. A minimum value of this parameter was searched for, since it accounts for a large percentage of the equipment operating cost.

An experimental strategy involving ninety study cases was established for the purpose of studying the effect that each of the factors mentioned may have on the response variables (*N* and *Q*). The experimental plan, obtained by the *D-optimal* strategy, contains the necessary number of experimental points required to obtain a representative response surface from the combinations of the levels of all factors. This experimental plan ensures that we fully study each factor within its range, not making the mistake of studying only a few points that may yield good results. The experimental plan is presented in Table 1. An Aspen Plus V7.1® simulator was used in the ninety study cases for obtaining the necessary information in the pre-design stage, as well as in the optimal design stage. In all cases, we used the RK-Aspen property method for predicting all phases. The Redlich-Kwong-Aspen equation-of-state is the basis for the RK-ASPEN property method and can be consulted at [18].

**Table 1:** Whole experimental plan with 90 study cases

**#**	***Fa***	***Fb***	***Fc***	***q***	***M***	**#**	***Fa***	***Fb***	***Fc***	***Q***	***M***	**#**	***Fa***	***Fb***	***Fc***	***q***	***M***
1	0.100	0.800	0.100	1.00	3	31	0.450	0.100	0.450	1.00	3	61	0.333	0.567	0.100	0.00	2
2	0.800	0.100	0.100	1.00	2	32	0.100	0.450	0.450	1.00	1	62	0.217	0.567	0.217	0.00	1
3	0.100	0.100	0.800	1.00	1	33	0.450	0.100	0.450	1.00	2	63	0.333	0.333	0.333	1.00	3
4	0.100	0.800	0.100	0.00	3	34	0.100	0.450	0.450	0.00	3	64	0.567	0.100	0.333	0.00	2
5	0.800	0.100	0.100	0.00	2	35	0.450	0.100	0.450	0.00	2	65	0.333	0.567	0.100	1.00	1
6	0.100	0.100	0.800	0.00	1	36	0.450	0.450	0.100	1.00	1	66	0.217	0.567	0.217	0.50	3
7	0.800	0.100	0.100	1.00	3	37	0.450	0.100	0.450	0.00	3	67	0.217	0.217	0.567	1.00	2
8	0.100	0.800	0.100	1.00	2	38	0.450	0.450	0.100	0.00	2	68	0.333	0.567	0.100	0.00	1
9	0.100	0.800	0.100	0.00	1	39	0.100	0.450	0.450	0.00	1	69	0.333	0.333	0.333	0.50	3
10	0.100	0.100	0.800	1.00	3	40	0.217	0.567	0.217	1.00	3	70	0.333	0.333	0.333	0.00	2
11	0.800	0.100	0.100	1.00	1	41	0.333	0.567	0.100	0.50	2	71	0.567	0.333	0.100	0.50	1
12	0.100	0.100	0.800	1.00	2	42	0.100	0.567	0.333	0.50	1	72	0.333	0.100	0.567	0.50	3
13	0.800	0.100	0.100	0.00	3	43	0.567	0.217	0.217	0.00	3	73	0.217	0.567	0.217	1.00	2
14	0.100	0.100	0.800	0.00	2	44	0.567	0.100	0.333	0.50	2	74	0.567	0.333	0.100	1.00	3
15	0.100	0.800	0.100	1.00	1	45	0.567	0.217	0.217	1.00	1	75	0.333	0.100	0.567	0.00	1
16	0.100	0.100	0.800	0.00	3	46	0.100	0.567	0.333	0.00	3	76	0.100	0.333	0.567	0.00	2
17	0.100	0.800	0.100	0.00	2	47	0.100	0.333	0.567	0.50	1	77	0.333	0.567	0.100	0.00	3
18	0.800	0.100	0.100	0.00	1	48	0.567	0.333	0.100	0.50	2	78	0.333	0.100	0.567	1.00	1
19	0.100	0.800	0.100	0.50	3	49	0.567	0.100	0.333	1.00	3	79	0.100	0.333	0.567	0.00	3
20	0.800	0.100	0.100	0.50	2	50	0.333	0.100	0.567	0.50	2	80	0.567	0.100	0.333	1.00	2
21	0.100	0.100	0.800	0.50	1	51	0.567	0.217	0.217	0.00	1	81	0.567	0.100	0.333	0.50	1
22	0.450	0.450	0.100	1.00	3	52	0.100	0.567	0.333	1.00	2	82	0.100	0.333	0.567	1.00	3
23	0.100	0.450	0.450	1.00	2	53	0.567	0.100	0.333	0.00	3	83	0.333	0.100	0.567	0.00	2
24	0.450	0.100	0.450	1.00	1	54	0.217	0.217	0.567	0.00	1	84	0.567	0.333	0.100	0.00	1
25	0.450	0.450	0.100	0.00	3	55	0.100	0.567	0.333	0.00	2	85	0.333	0.100	0.567	1.00	3
26	0.100	0.450	0.450	0.00	2	56	0.100	0.567	0.333	1.00	3	86	0.333	0.333	0.333	0.25	1
27	0.450	0.100	0.450	0.00	1	57	0.217	0.217	0.567	1.00	1	87	0.333	0.333	0.333	0.75	2
28	0.100	0.450	0.450	1.00	3	58	0.567	0.217	0.217	1.00	2	88	0.800	0.100	0.100	0.50	3
29	0.450	0.450	0.100	1.00	2	59	0.217	0.567	0.217	1.00	1	89	0.333	0.333	0.333	0.75	1
30	0.450	0.450	0.100	0.00	1	60	0.333	0.333	0.333	0.00	3	90	0.100	0.450	0.450	0.50	2

## Results and discussion

Table 2 presents the results obtained for the number of theoretical stages and the thermal load when both algorithms were applied in the design of Petlyuk columns. Feasible results were obtained with the proposed method (Method 2) for all 90 study cases with a solution that met all the established specifications, whereas the traditional method (Method 1) provided feasible results only for 8 of the 90 study cases.

**Table 2:** Results obtained applying both algorithms for the pre-design of Petlyuk columns

#	**Method 1**		**Method 2**		**#**	**Method 1**		**Method 2**		**#**	**Method 1**		**Method 2**	
	***N***	***Q***	***N***	***Q***		***N***	***Q***	***N***	***Q***		***N***	***Q***	***N***	***Q***
1	--	--	134	203.48	31	--	--	153	10.04	61	39	4.10	50	4.31
2	--	--	76	4.01	32	32	3.49	53	2.51	62	--	--	43	4.18
3	32	4.28	51	7.83	33	39	2.47	65	18.94	63	--	--	136	21.57
4	57	7.43	132	14.23	34	--	--	98	27.27	64	38	4.36	57	12.32
5	38	5.48	70	7.74	35	--	--	59	11.74	65	--	--	58	3.40
6	--	--	50	10.10	36	--	--	48	3.37	66	--	--	128	54.58
7	--	--	159	22.05	37	--	--	131	35.11	67	38	4.65	61	4.04
8	--	--	61	5.83	38	40	6.46	65	4.16	68	--	--	44	5.19
9	--	--	40	12.13	39	--	--	51	6.32	69	--	--	138	13.11
10	--	--	101	18.21	40	--	--	129	58.20	70	39	6.06	52	9.67
11	--	--	67	3.52	41	41	4.06	58	4.34	71	39	4.11	61	3.60
12	--	--	60	22.20	42	32	4.04	53	2.59	72	--	--	124	25.46
13	--	--	154	15.52	43	--	--	136	18.43	73	39	4.29	63	8.68
14	--	--	56	50.65	44	38	4.58	61	9.07	74	--	--	147	19.88
15	--	--	41	5.78	45	--	--	54	10.15	75	--	--	50	30.66
16	--	--	94	636.37	46	--	--	116	80.44	76	--	--	58	14.70
17	36	5.59	46	13.50	47	30	6.15	52	27.25	77	--	--	130	55.14
18	--	--	60	4.29	48	--	--	68	4.45	78	39	1.60	55	3.81
19	--	--	134	14.11	49	--	--	147	32.57	79	--	--	104	151.02
20	--	--	73	4.70	50	40	3.09	61	9.77	80	39	2.77	64	6.34
21	--	--	50	19.50	51	36	4.02	51	5.98	81	37	2.98	53	5.35
22	--	--	136	38.46	52	36	4.60	61	3.41	82	--	--	102	21.19
23	37	6.95	61	4.99	53	--	--	139	10.20	83	37	0.41	59	22.88
24	37	1.69	55	5.38	54	--	--	50	11.41	84	38	4.29	59	3.90
25	--	--	135	33.19	55	35	8.95	59	5.26	85	--	--	137	6.39
26	--	--	59	14.44	56	--	--	132	13.00	86	35	2.92	46	5.70
27	34	2.76	48	8.29	57	36	2.34	53	2.09	87	42	3.16	63	2.26
28	--	--	128	10.79	58	--	--	68	3.16	88	--	--	156	15.71
29	--	--	66	3.78	59	34	3.69	55	3.72	89	36	2.63	56	3.09
30	37	1.69	46	5.88	60	--	--	137	17.04	90	36	7.64	59	4.67

**Figure 5:**
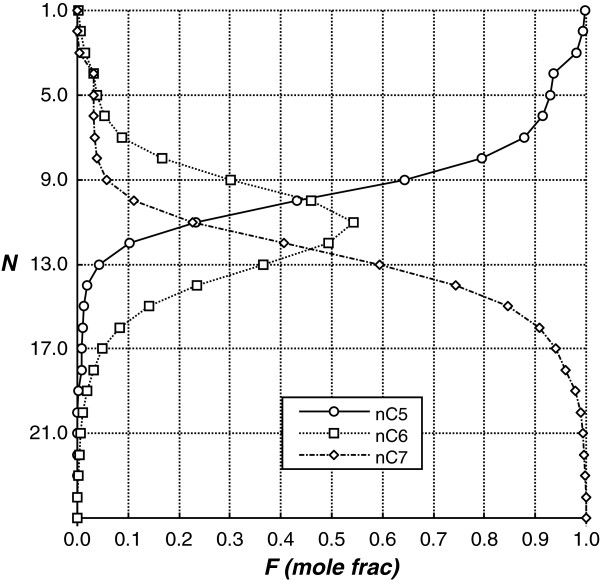
**Composition profiles obtained for the main column by the traditional pre-design method.** Study case 27: n-pentane/n-hexane/n-heptane, *Fa* = 0.45, *Fb* = 0.10, *Fc* = 0.45 and *q* = 0.0.

An analysis of the failures of Method 1 is presented. Table 3 presents a summary of the results obtained with Method 1. Only 8 study cases (which represent 8.9% of the study cases involved) obtained feasible solutions that met all the established specifications of the problem addressed (type A solution), 5 of which are for the *M*_*1*_ mixture, 3 for the *M*_*2*_ mixture, and none for the *M*_*3*_ mixture. This explains, perhaps, why in previous works with Petlyuk columns only ideal mixtures have been used.Other feasible solutions were obtained for 28 study cases (type B and C solution), but the results were different from those required (type 2 solutions). For example, the type B solution implies that purities lower than 98% are obtained in a component by applying the design method, even when a value of 98% had been established. The type C solution involves a failure in the purity of two components. Figure 5 shows this for study case number 27 (type B solution), in which a purity of 55% was obtained for the intermediate component (n-hexane). Infeasible solutions were obtained for 54 study cases (60% of cases), that is to say, the FUG equations did not provide a solution for the conditions specified in the traditional method.

**Table 3:** Results with feasible solutions obtained with the traditional pre-design method, which uses fixed fractions

**Solution type**	**Description of the solution type**	**Case of study number (see Table**1**)**
**A**	Feasible solution with all satisfied specifications	5, 32, 51, 70, 71, 80, 84, 89
**B**	Feasible solution with two output purity specifications satisfied	3, 24, 27, 33, 42, 44, 47, 50, 52, 55, 57, 59, 64, 67, 73, 78, 81, 86, 87
**C**	Feasible solution with one output purity specification satisfied	4, 17, 23, 30, 38, 41, 61, 83, 90
**D**	Infeasible solution. There is no convergence in the pre-design stage	1, 2, 6, 7, 8, 9 10, 11, 12, 13, 14, 15, 16, 18, 19, 20, 21, 22, 25, 26, 28, 29, 31, 34, 35, 36, 37,39, 40, 43, 45, 46, 48, 49, 53, 54, 56, 58, 60, 62, 63, 65, 66, 68, 69, 72, 74, 75, 76, 77, 79, 82, 85, 88

In all cases where type A, B and C solutions were obtained, the number of theoretical stages in the traditional method was less that in the method proposed here. Although this is an advantage of the traditional method, the proposed method always converges to the desired purity specifications. Comparing these two aspects, the proposed method turns out to be the better one, since the design of the Petlyuk columns is based on the purity of the products. With respect to the thermal load, similar results were obtained with both methods; in some cases the proposed method yielded better results than the traditional method and in other cases the reverse occurred. These results show that the proposed method is comparable to the traditional method in terms of the economic benefits reported in other studies, but it provides feasible results always. Thus, the proposed method allows for exploring a number of conditions, which was not possible with the traditional method.

Tables 4 and 5 present the descriptive statistics of the results obtained through the proposed method (Method 2), regarding the number of stages and the thermal load, grouped by each of the mixtures. In these tables, it can be seen that the *M*_*1*_ mixture is easier to separate and that the *M*_*3*_ mixture is the hardest to separate, since the *M*_*1*_ mixture has lower values of *N* as *Q* and the *M*_*3*_ mixture has much higher values.

**Table 4:** **Statistics of number of theoretical stages ( *N *) obtained by proposed method, grouped by mixture**

**Mixture**	**Min.**	**Mean**	**Max.**
***M***_***1***_	40	51.77	67
***M***_***2***_	46	61.30	76
***M***_***3***_	94	130.90	159
**Total**	**40**	**81.32**	**159**

**Table 5:** **Statistics of energy consumption (*Q *** **in MW) obtained by proposed method, grouped by mixture**

**Mixture**	**Min.**	**Mean**	**Max.**
***M***_***1***_	2.09	7.57	30.66
***M***_***2***_	2.26	9.87	50.37
***M***_***3***_	6.39	56.43	636.37
**Total**	**2.09**	**24.62**	**636.37**

Since the proposed method (Method 2) was adequate in all studied cases, an analysis of variance (ANOVA) and a response surface analysis were performed using a quadratic model for the mixed factors and a linear model for the process factors. In this case, we used an ANOVA, since the response, obtained from simulations, had a certain degree of bias, as it came from optimization results (Equations 1, 2 and 3) that are sensitive to initial conditions. For example, *N* depends on the composition of the flows of the interconnection streams that were obtained in the pre-design stage, established by optimization. This ANOVA is presented only to explore the response surface of variables *N* and *Q*. Table 6 presents a summary of the ANOVA performed with transformations for the number of stages and the thermal load. In both cases, the model is statistically significant (*p* < 0.05) with respect to the response and represents a good adjustment, since the coefficient of determination (*R*^*2*^) is in reasonable agreement with the adjusted coefficient of determination (*R*^*2*^_*adj*_); therefore, these models can be used to navigate the design space. This provides us with a safe way of representing the response surfaces with respect to the number of stages and the thermal load.

**Table 6:** Statistics by response (number of stages and thermal load) obtained while applying an ANOVA

**Variable**	**Transf.**	**SSE**	**SSR**	**df**	**F-value**	***p-value***	***R***^**2**^	***R***^**2**^_***adj***_
***N***	Sqrt.	3.99	335.31	23	241.10	<0.0001	0.9882	0.9841
***Q***	Inv. Sqrt.	0.49	1.48	23	8.68	<0.0001	0.7515	0.6649

**Figure 6:**
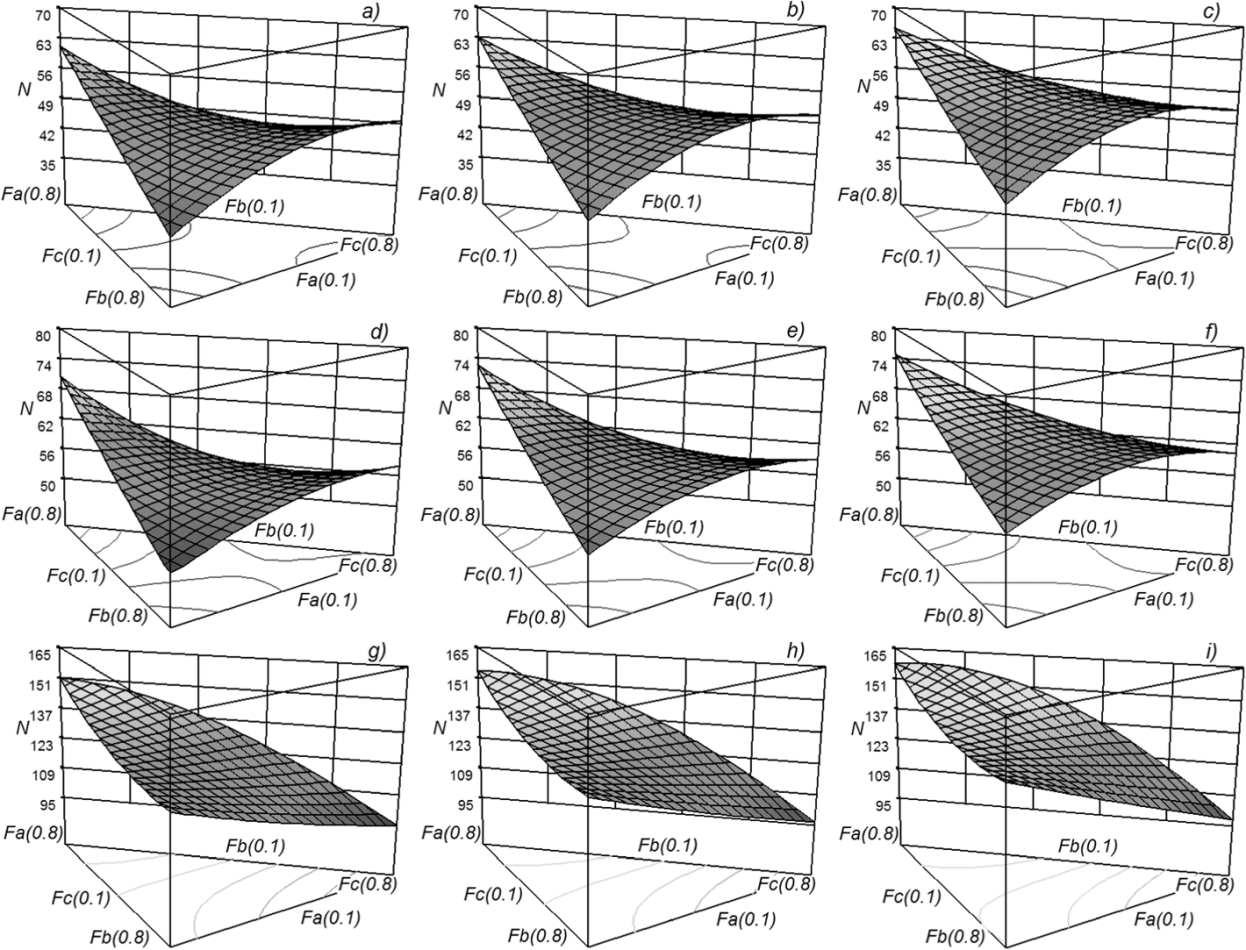
**Response surface for the N variable as a function of the composition of the feed.** Each subfigure represents a combination of the type of mixture with the thermal conditions of the feed. The rows represent the mixture (*M*_*1*_, *M*_*2*_, *M*_*3*_) and the columns represent the feed thermal conditions: saturated vapour (subfigures **a**, **d** and **g**), vapour-liquid mixture with *q* = 0.5 (subfigures **b**, **e** and **h**) and saturated liquid (subfigures **c**, **f** and **i**).

**Figure 7:**
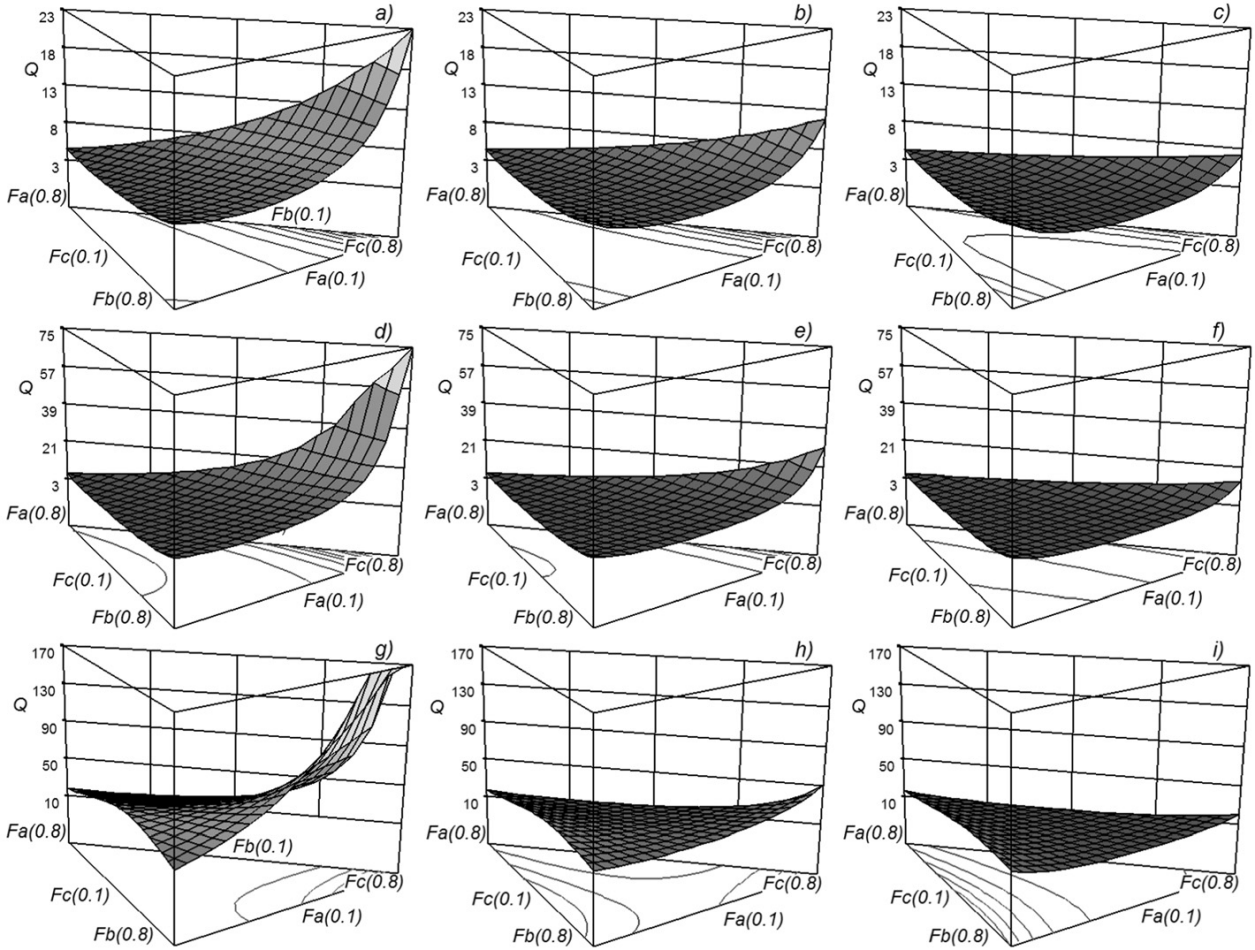
**Response surface for the Q (MW) variable as a function of the feed composition.** Each subfigure represents a combination of the type of mixture with the thermal conditions of the feed. The rows represent the mixture (*M*_*1*_, *M*_*2*_, *M*_*3*_) and the columns represent the feed thermal conditions: saturated vapour (subfigures **a**, **d** and **g**), vapour-liquid mixture with *q* = 0.5 (subfigures **b**, **e** and **h**) and saturated liquid (subfigures **c**, **f** and **i**).

It can be observed in Figure 6 that when the light component (component A) is found in greater quantity, there is a larger number of theoretical stages and therefore a higher cost in equipment. However, a lower number of stages, and hence a lower cost in equipment, is achieved when the intermediate component is found in a larger quantity only with the *M*_*1*_ and *M*_*2*_ mixtures (subfigures *a-f*), while the same happens with the *M*_*3*_ mixture when there is a larger quantity of the heavy component (subfigures *g-i*). With the same mixture, when the thermal conditions of the feed change, the qualitative form and the magnitude order of the response surface do not suffer any considerable changes. In this way, the cost of the Petlyuk column is little sensitive to changes in the thermal conditions of the feed. For the same thermal conditions of the feed, when the mixture changes the response surface undergoes considerable changes, qualitative as well as quantitative. This implies that the capital cost of a Petlyuk column is sensitive to the type of mixture that one is trying to separate. Mixture *M*_*3*_ is more difficult to separate (subfigures *g-i*).Figure 7 shows that a greater thermal load, and therefore a higher operation cost, occurs when the heavy component is found in greater proportion, while a smaller thermal load occurs when the lighter or intermediate component is found in greater proportion. In all mixtures, when the thermal conditions of the feed change, the qualitative form of the response surface does not suffer considerable changes. However, when the thermal condition of the feed changes, the order of magnitude of the 5response suffers considerable changes too, which indicates that the operating cost of a Petlyuk column is very sensitive to changes in this factor. For the same thermal conditions of the feed, the response surface undergoes considerable quantitative changes when the mixture changes. That is why the cost of operation of a Petlyuk column is very sensitive to the different mixtures that one is trying to separate.

## Conclusions

The proposed strategy allow us not only to find a feasible design that meets output specifications but also one in which energy consumption is comparable to that obtained by the traditional pre-design model, which uses fixed fractions. We are certain that with this method it is possible to obtain feasible solutions for a wide range of conditions, allowing for more general studies on the design of distillation columns of the Petlyuk-type. We are contemplating conducting a larger study taking into account other process parameters (e.g. the reflux ratio).

### Nomenclature

*df* Degree of freedom

*Fa* Molar fraction of the intermediate volatile component kgmol A/kgmol

*Fb* Molar fraction of the most volatile component kgmol B/kgmol

*Fc* Molar fraction of the least volatile component kgmol C/kgmol

F*-value* Fisher value

*M* Type of mixture

*M*_*1*_ n-pentane/n-hexane/n-heptane mixture

*M*_*2*_ n-hexane/n-heptane/n-octane mixture

*M*_*3*_ n-hexane/benzene/toluene

*N* Number of theoretical stages

*p-value* Probability value

*q* Thermal condition of the feed

*Q* Thermal load MW

*Q*_*c*_ Heat removed in the condenser MW

*Q*_*r*_ heat required in the reboiler MW

*R*^2^ Coefficient of determination

*R*^2^_adj_ Adjusted coefficient of determination

SD Standard deviation

SSE Sum square error

SSR Sum square regression

## Competing interests

Each author of this paper declares no competing interests of any sort with the brands and companies cited in the text. Furthermore, the authors are/were not employed by any of the cited companies and have not received fees for consulting or research funding from the cited brands.

## Authors’ contributions

JGZS carried out the calculation and drafted the manuscript. RGG, JBF and JGZS analyzed the results. RGG, JBF and JGZS corrected the English expressions. All authors have read and approved the final manuscript.

## References

[B1] Petlyuk FB Platanov VM Slavinskii DM Thermodynamically optimal method for separating multicomponent mixtures Int Chem Eng 1965 5 555 561

[B2] Kaibel G Distillation column with vertical partitions Chem Ing Techn 1987 10 92 98

[B3] Hairston D The divide in distillation Chem Eng 1999 106 32 35

[B4] Yeomans H Grossmann IE Optimal design of complex distillation columns using rigorous tray-by-tray disjunctive programming models Ind Eng Chem Res 2000 39 4326 4335 10.1021/ie0001974

[B5] Rév E Emitir M Szitkai Z Fonyó Z Energy savings of integrated and coupled distillation systems Comput Chem Eng 2001 25 119 140 10.1016/S0098-1354(00)00643-8

[B6] Gutiérrez-Antonio C Briones-Ramírez A Pareto front of ideal Petlyuk sequences using a multiobjective genetic algorithm with constraints Comput Chem Eng 2009 33 454 464 10.1016/j.compchemeng.2008.11.004

[B7] Ramírez-Corona N Jiménez-Gutiérrez A Castro-Agüero A Rico-Ramírez V Optimum design of Petlyuk and divided-wall distillation systems using a shortcut model Chem Eng Res Des 2010 88 1405 1418 10.1016/j.cherd.2010.02.020

[B8] Gutiérrez-Antonio C Briones-Ramírez A Jiménez-Gutiérrez A Optimization of Petlyuk sequences using a multi objective genetic algorithm with constraints Comput Chem Eng 2011 35 236 244 10.1016/j.compchemeng.2010.10.007

[B9] Tedder DW Rudd DF Parametric studies in industrial distillation: part I Design Comparisons AIChE J 1978 24 303 315 10.1002/aic.690240220

[B10] Glinos KN Malone MF Optimality regions for complex column alternatives in distillation systems Chem Eng Res Des 1988 66 229 240

[B11] Carlberg N Westerberg W Temperature-heat diagrams for complex column. 2. Underwood´s method side strippers and enrichers Ind Eng Chem Res 1989 28 1379 1386 10.1021/ie00093a017

[B12] Agrawal R Synthesis of distillation columns configurations for a multi-component separation Ind Eng Chem Res 1996 35 1059 1071 10.1021/ie950323h

[B13] Christiansen AC Skogestad S Lien K Complex distillation arrangements: extending the petlyuk ideas Comput Chem Eng 1997 21 Suppl S237 S242

[B14] Halvorsen IJ Skogestad S Minimum energy consumption in multicomponent distillation. 1. V_min_ Diagram for two-product columnnd Eng Chem Res 2003 42 596 604 10.1021/ie010863g

[B15] Halvorsen IJ Skogestad S Shortcut analysis of optimal operation of Petlyuk distillation Ind Eng Chem Res 2004 43 3994 3999 10.1021/ie034177o

[B16] Seader JD Henley EJ Roper DK Separation Process Principles- Chemical and Biochemical Operations 2010 New York: John Wiley and Sons

[B17] Blancarte-Palacios JL Bautista-Valadés MN Hernández-Castro S Rico-Ramírez V Jiménez-Gutiérrez A Energy-efficient design of thermally coupled distillation sequences for four-component mixtures Ind Eng Chem Res 2003 42 5157 5164 10.1021/ie030297k

[B18] Mathias PM A versatile phase equilibrium equation-of-state Ind Eng Chem Process Des Dev 1983 22 385 391 10.1021/i200022a008

